# Supplementary material for: Leptospira enrichment culture followed by ONT metagenomic sequencing allows better detection of Leptospira presence and diversity in water and soil samples
Source: PLoS Negl Trop Dis. 2022 Oct 26;16(10):e0010589. doi: 10.1371/journal.pntd.0010589 (PMC9639851; doi:10.1371/journal.pntd.0010589)
Supplement: S1 File — The bar charts grouped here represent the levels (0–4) of Leptospira growth in water(A) and soil (B) cultures over a period of four weeks. Each bar chart displays the growth for one of the four medias used along with the different selective antimicrobials added to some cultures. (A: EMJH Media, B: Fletcher, C: Korthof, D: Stuart) (DOCX) [file pntd.0010589.s001.docx]

**S1 File**

**Growth of *Leptospira* like organisms in various cultures. The bar charts grouped here represent the levels (0-4) of *Leptospira* growth in water(A) and soil (B) cultures over a period of four weeks. Each bar chart displays the growth for one of the four medias used along with the different selective antimicrobials added to some cultures. (A: EMJH Media, B: Fletcher, C: Korthof, D: Stuart)**

A


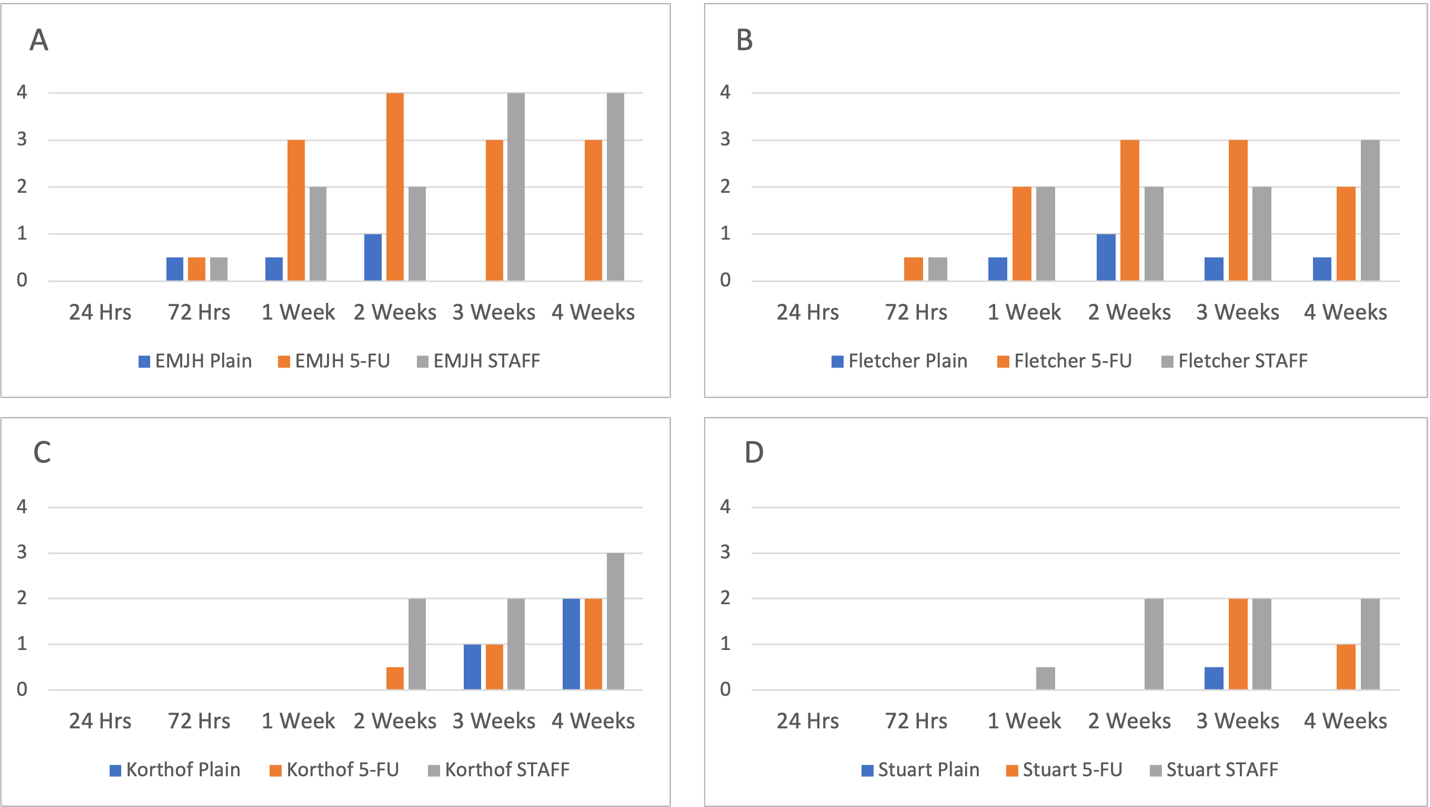


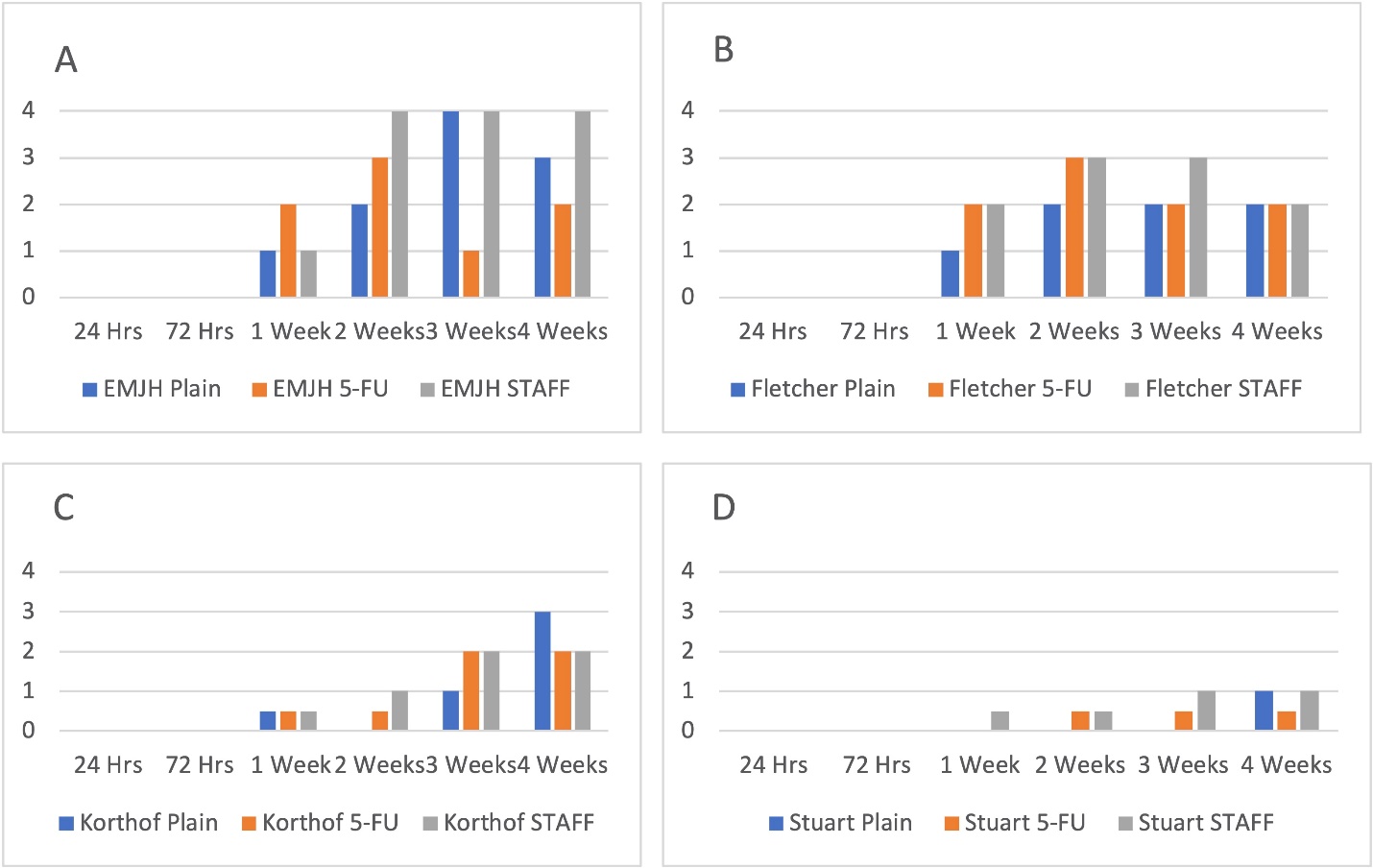


B
