# Supplementary material for: Leptospira enrichment culture followed by ONT metagenomic sequencing allows better detection of Leptospira presence and diversity in water and soil samples
Source: PLoS Negl Trop Dis. 2022 Oct 26;16(10):e0010589. doi: 10.1371/journal.pntd.0010589 (PMC9639851; doi:10.1371/journal.pntd.0010589)

**S2 File: PCR results from enrichment cultures**

**A representative result graph of PCR on enrichment cultures: Bar charts display the Cq values of soil A) and water (B) cultures tested after 4 weeks of incubation. Sample names along the x axis are types of media used to culture samples and the selective antimicrobial treatment. Cultures were tested with real-time PCR using *lipl32* and *16s* gene markers.**

A


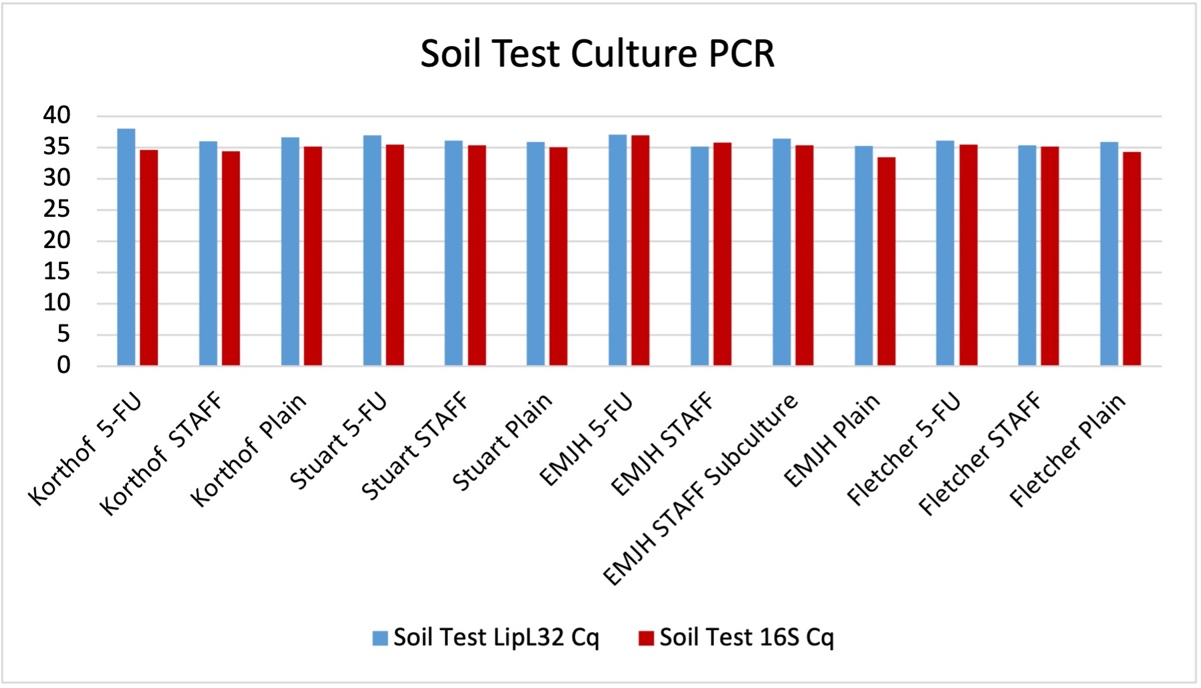


B


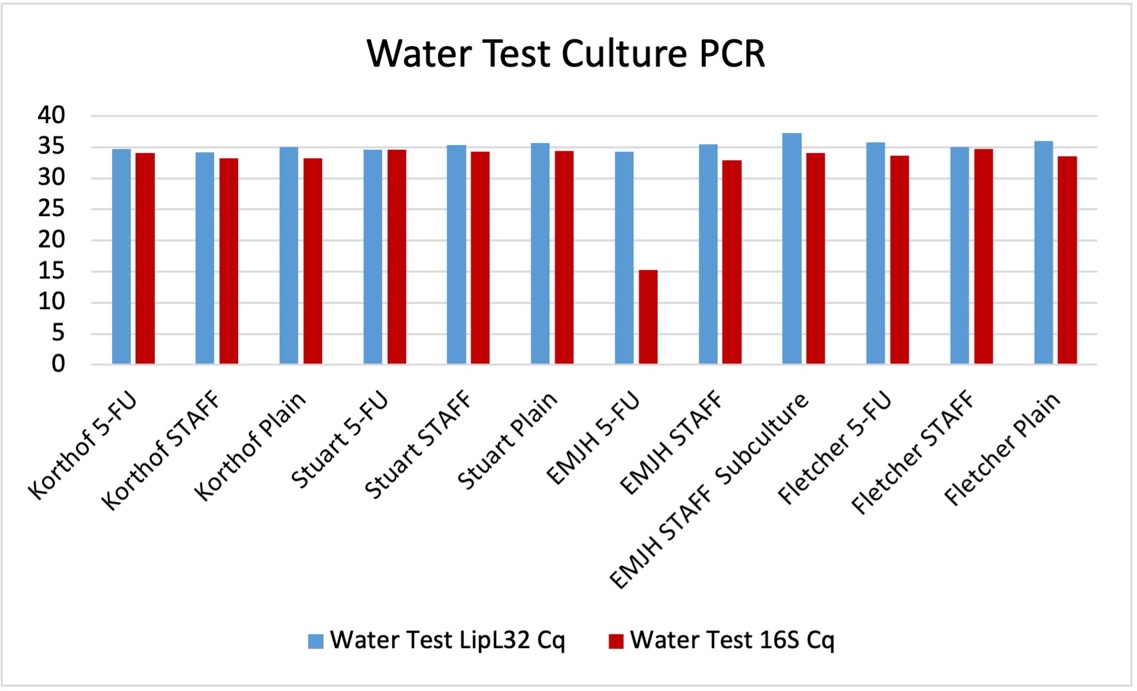

Supplement: S2 File — A representative result graph of PCR on enrichment cultures: Bar charts display the Cq values of soil (A) and water (B) cultures tested after 4 weeks of incubation. Sample names along the x axis are types of media used to culture samples and the selective antimicrobial treatment. Cultures were tested with real-time PCR using lipL32 and 16sRNA gene markers. (DOCX) [file pntd.0010589.s002.docx]
