# Supplementary material for: Leptospira enrichment culture followed by ONT metagenomic sequencing allows better detection of Leptospira presence and diversity in water and soil samples
Source: PLoS Negl Trop Dis. 2022 Oct 26;16(10):e0010589. doi: 10.1371/journal.pntd.0010589 (PMC9639851; doi:10.1371/journal.pntd.0010589)
Supplement: S3 File — Highlighted in blue and pointed to by a red arrow are Leptospira reads collected from soil (A) and water (B) samples in this study. Number labelled next to each node is the bootstrap support value for this specific node. Please note that L. macculloughii was a misidentified species from the mixed culture of L. meyeri and L. levetti (24). Therefore, in the Figs 2 and 3, Reads classified as L. macculloughii was grouped together with the unclassified Leptospira spp. (DOCX) [file pntd.0010589.s003.docx]

**S3 File**

**Phylogenetic tree showing position of *Leptospira* classification from 16S dataset. Highlighted in blue and pointed to by a red arrow are *Leptospira* reads collected from soil (A) and water (B) samples in this study. Number labelled next to each node is the bootstrap support value for this specific node. Please note that *L. macculloughii*  was a misidentified species from the mixed culture of *L. meyeri* and *L. levetti* [1].**

1. Soil samples


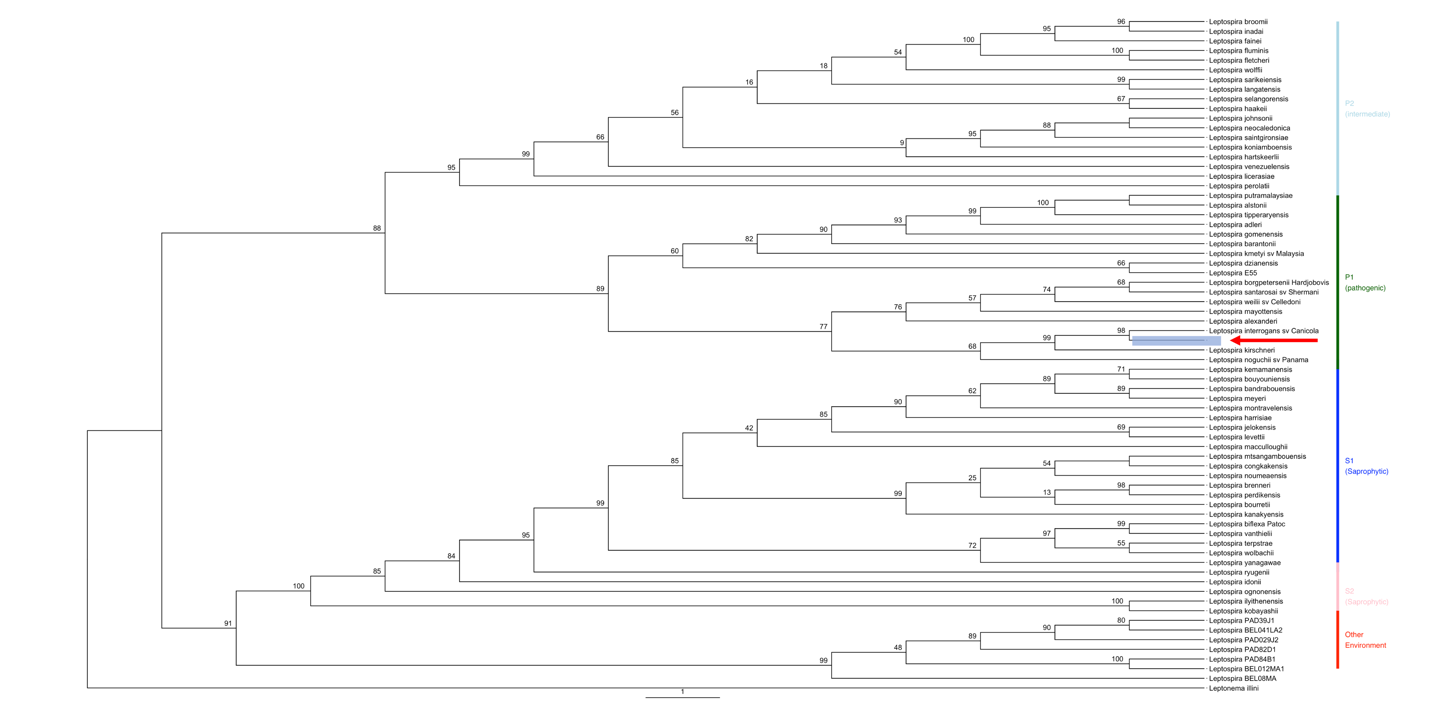


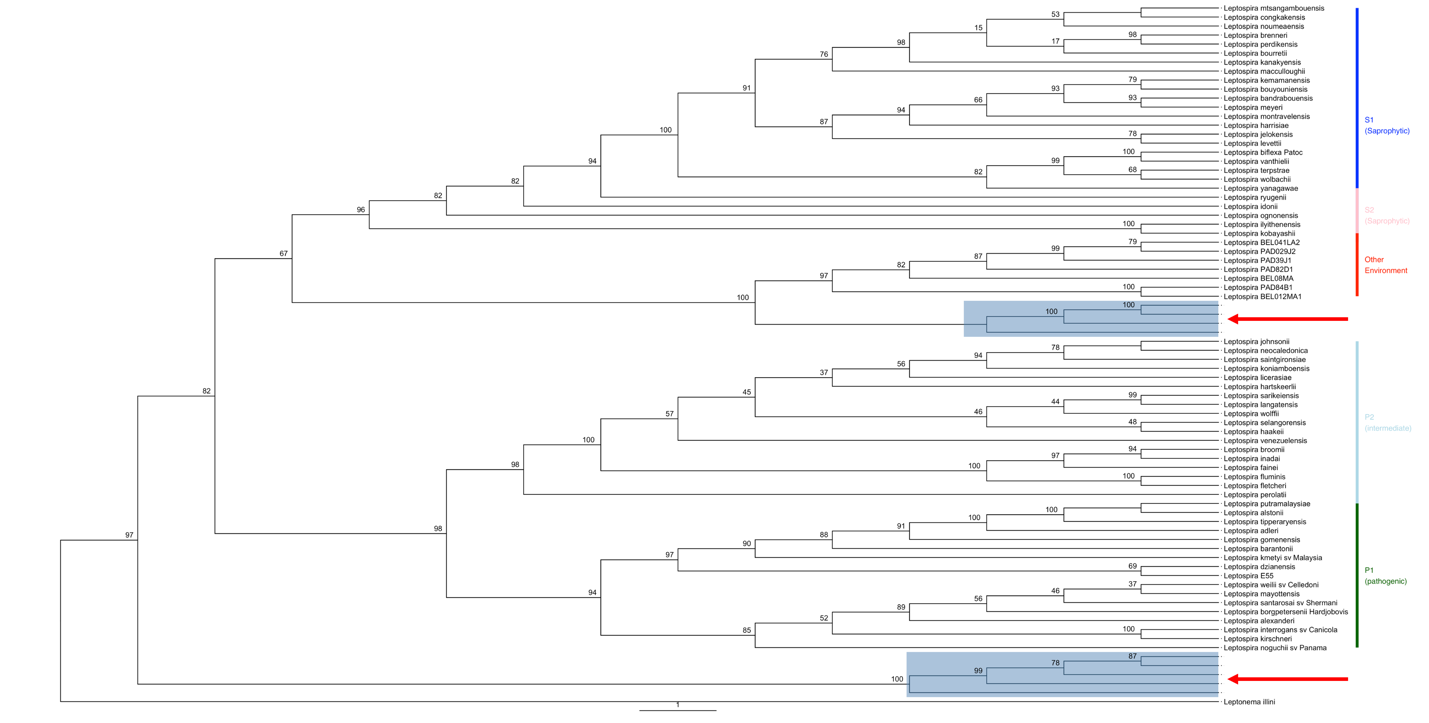


B- Water samples

References

1. Thibeaux R, Iraola G, Ferrés I, Bierque E, Girault D, Soupé-Gilbert M-E, et al. Deciphering the unexplored Leptospira diversity from soils uncovers genomic evolution to virulence. Microbial Genomics. 2018;4. doi:10.1099/mgen.0.000144
