# Supplementary material for: Leptospira enrichment culture followed by ONT metagenomic sequencing allows better detection of Leptospira presence and diversity in water and soil samples
Source: PLoS Negl Trop Dis. 2022 Oct 26;16(10):e0010589. doi: 10.1371/journal.pntd.0010589 (PMC9639851; doi:10.1371/journal.pntd.0010589)
Supplement: Attachment — Submitted filename: Reply_to_Reviewer_Comments_R1.docx [file pntd.0010589.s004.docx]

**Reply to reviewers’ comments**

**Reviewer 1**

We thank reviewer 1 for the time and effort it took to review our manuscript and for acknowledging the importance of this work.

**Reviewer #2:**

We wish to thank reviewer 2 for the time and effort taken to critique this manuscript. We have attempted to address all of the comments raised by the reviewer whenever possible.

1. It would be good to have a picture of the study site. Further detailing of the study site will be helpful.

The study is a method comparison, not a surveillance study; samples were collected from a single site. A map may not add any valuable information to the manuscript.

2. Line 101: It is mentioned that 50g of the soil sample was collected. But in the Fig 1 and this line, it says 25g. Please clarify.

The original collection was about one liter of water and 50 g of soil. But we only processed a part of it. We acknowledge the confusion, changed the wording and removed the initial collection quantity to improve clarity.

3. Line 112: I don't understand the rationale for adding 10^7 Leptospira/ml in the control sample.

We are not clear about this concern raised by the reviewer. We spiked the samples with a standardized *Leptospira* inoculum before the downstream processing.

Testing spiked samples is a part of the microbiology quality control procedures. We believe it is important to use this control to verify whether our methods were working.

4. Please add the primer sequences in a separate table.

Primer sequences are from published articles, and are available in the referenced publications.

5. Why was the cut off set at 40? This is extremely high for analysis. Please clarify.

The cut-off 40-45 is the standard Cycle Threshold value used in real-time PCR reactions. It is a widely accepted cutoff point.

6. Line 274: Mention full form of NJ

We used a Maximum Likelihood analysis and is updated in the manuscript.

7. The Fig 3 description is coming before figure 2 in the text. Please make sure all the figures numbers and description come in succession. If needed, re format the whole manuscript so that this is followed. It made the manuscript very difficult to read.

In the revised manuscript, figure captions are consistently presented.

8. This above point is also applicable for all the Tables. Please re format the manuscript.

Same as above.

9. Line 163: Was MAT tried for the samples? Please clarify why it wasn't used for Leptospira detection.

MAT is a test used for detecting antibodies after infection or exposure or for serologic characterization of *Leptospira* isolates using antibodies generated against *Leptospira* serovars. Our study used soil and water samples, and hence performing MAT would not be possible or applicable in this study.

1. Fig 1 relating to the methods is very simple. Please re draw it in a better way with more details for clarity.

Although we attempted to redo this figure, we decided to remove this figure from the manuscript due to uncertainty in copyright concerns raised by the journal upon resubmission. We have explained the process clearly in the materials and methods.

2. Fig 2 has 16 and 38 on top of the first bars. What does this mean? Please clarify.

These are CT values, and we acknowledge the confusion. This issue has been addressed in the revised version.

3. What is the implication for not getting lipl32 in the soil test and water test? Please explain. Why do the authors think that this may be the case?

*Lipl 32* is a gene marker commonly used in PCR for detecting the presence of pathogenic *Leptospira*. In addition to the absence of *Leptospira*, there are multiple reasons for not detecting it, which is elaborated in the discussion Most likely at least in our study, the absence potentially correlates with sensitivity issues of the test and PCR inhibition.

4. Figure 3 and 4 are of very bad quality. Please use high resolution images.

We have submitted high-resolution images tested using the PLOS diagnostic tool in the revised manuscript.

5. The phylogenetic trees in the supporting information should have bootstrapping values on the axes.

We performed 1000 bootstrapping analyses on the new maximum likelihood phylogeny reconstructed using Leptospiral 16S rRNA reads obtains from this study with the reference 16S rRNA sequences obtained from the SILVA database. The bootstrap values between *leptospiral* reads obtained from this study had over 90% support value in all the phylogenetic relationships we observed in this study. These also include the phylogeny with *leptospiral* reads obtained from the soil sample, which has bootstrap support of 99% on clades with pathogenic species *L. interrogans* and *L. kirschneri*.

6. Why do the authors think the trend of Leptospira growth was different for the different media as shown in S2?

The media we tested are commonly used for growing *Leptospira* and differ in ingredients and nutrients that support *Leptospira* growth. In addition, the nutrient requirement for each species may differ. These facts are well known in the *Leptospira* field.

Conclusions

Reviewer #2:

1. The discussion section doesn't adequately address the significance of the results obtained.

The significance of the study is emphasized in many areas in the manuscript

2. The public health relevance hasn't been addressed well.
The public health relevance of the study is emphasized Introduction and Discussion

Reviewer #2: The figures are of very low quality.

The revised manuscript has images with the required resolution.

Summary and General Comments

Reviewer #2: The authors present an interesting study but the manuscript is not well edited and formatted. The Fig numbers are not in succession and the English needs to be proof read by a native English speaker. Also, the discussion section doesn't adequately address the significance of the results obtained. The manuscript needs major work to address these concerns.

All of the comments are addressed when possible. We have carefully reviewed the manuscript and attempted to edit and reformat the manuscript. All authors are proficient in the English language, and the first author is a “native speaker”.

Reviewer #3:

- It is known that 16S sometimes produces ambiguous results in terms of species classification, and this has been mentioned in several articles focusing on Leptospira classification. In this regard, it would have been good to try alternative targets as well. In a recent publication, Wilkinson and collaborators (2021) used glmU to detect specific species in culture and culture-independent samples, also using Nanopore. Although restricted to a few species, they obtained good results.

We agree with the reviewer. Many single targets have these issues in detecting the diversity of bacterial species. However, enrichment culture with metagenomic revealed a large diversity and is probably the way to go. 16s-based metagenomics is widely exploited in studying microbial diversity and the point we want to make is that this method in fact underestimates the microbial diversity especially when there is a low level of certain bacteria.

- The classification as pathogenic and intermediates is becoming obsolete. It may be pertinent to update to the more recent classification (P1, P2, S1, S2) in some parts of the document.
We have updated this in the revised version.

- Line 314-315 “Figure 4 summarizes microbial classification profiles of direct sequencing results”. It should refer to “enrichment and sequencing” instead of “direct sequencing results”.

Addressed in the revised version.

- The “Accession” column in Table 2 is empty.

Addressed in the revised version.

- The supplementary figure S1 and Figure 1 could be better designed to include the different samples and replicates as compared to just repeating the text. I am not sure are the replicates have been used.

Since the samples were collected only from a single location, we used only technical replicates. Although we attempted to redo this figure, we decided to remove these figures, due to uncertainty in copyright concerns raised by the journal upon resubmission. We have explained the process clearly in the materials and methods.

Reviewer #3: The Discussion is clearly too long as compared to the rest of the document and tend to over-interpret the data.

The discussion is of standard length for the study described here, and the authors have taken precautions not to overinterpret the data. We reevaluated the discussion and addressed any redundancy.

- Lines 370-374 “Our methodology used a double filtration system to improve the efficacy. First, we used a 40 μm filter to catch large debris that could block the smaller filter and impede filtration. Then the resulting filtrate was allowed to pass through a 0.45 μm filter to remove larger bacteria, assuming that Leptospira with a width of 0.1 μm would pass through the filter”. Double filtration is not described in methodology.

This is a sequential filtration using two types of filters to maximize Leptospira recovery and minimize contamination. We have clarified this in the revised version.

- Lines 376-378 “Unlike the direct PCR, culture enrichment followed by PCR could detect Leptospira DNA in these samples”. I find it relevant to show the Cq graphs for PCR on enriched cultures.

The results are now presented in the supplementary files in the revised version.

Lines 89-92 Although the amount of water/soil seems to be quite sufficient for downstream processing, it is not clear whether both types of samples were taken from a single point. In that case, sampling from several spots may (or may not) disclose more diversity in the Leptospira species detected.

The samples were taken from a single location at a single time point. We agree with the reviewer. The evaluation of more samples might detect more diverse species of *Leptospira*, but it is cost prohibitive. This study was a method evaluation and the large diversity of *Leptospira* was indeed an unexpected finding. We plan to conduct surveillance studies in multiple geographic locations as funding becomes available.

- Lines 98-99 “We spiked one of the 100 mL aliquots with Leptospira interrogans serovar Copenhageni (107 bacteria per mL) to use as the control, and the second aliquot was designated as the test sample”. It is not clear whether the specified concentration is that of the initial inoculum or the final concentration in the 100 mL aliquot.

It is the final concentration - this concentration was used to make sure that we can detect the presence even after multiple downstream procedures. It is clarified in the revised version.

- Lines 99-100 “The samples were further divided into 50 mL aliquots for PCR, sequencing, and culturing. A 100 mL sample was divided into three 50 mL aliquots”. How is that? I assume that one tube was used for PCR and sequencing, while the other was used for culture, it may be more specific.

We corrected this mistake in the revised version. Thank you for noticing it

- Lines 101-102 “For the processing of soil samples, the 25 g of soil was divided between two flasks and then mixed with 100 mL of phosphate-buffered saline (PBS)”. It was previously stated that 50 g were taken. So, was only half used or were 25g/25g processed in parallel?

In the revised version, we have improved the clarity of this description.

- Lines 107-108 “We spiked one of the aliquots with Leptospira interrogans serovar Copenhageni (107 bacteria per-mL) and designated it as "control"”. Again, it is not clear whether this was the concentration of the initial culture or the concentration in the 75 mL.

It is the final concentration.

- Lines 175-176 “A composite of positive samples of culture and soil was used to reduce the cost of testing”. It is unclear if the positive cultures from water and soil samples were mixed, or the positive culture from the water alone, and the positive soil samples separately.

The composites were made separately for water and soil.
--------------------
Reviewer #3: Direct PCR results from environmental samples

- Lines 234-236 Although the authors acknowledge that the results for 23S are controversial, as they are detected in the tested soil, and the spiked water, but not in the spiked soil and the tested water, it is not explained why this could happen. I fear an exchange between tested soil and tested water samples. That would make more sense. On the other hand, I assume the variation in Cq is due to technical replicates in a single qPCR run, is that correct? Did the authors perform the qPCR more than once, in independent experiments?

We are confident that there is no switching of samples as we use strict quality control during sample processing, and extraction and PCRs are not done simultaneously. The PCR was performed in duplicate for each sample and the Cq values for the run were averaged together.

- As for Figure 2 also, considering they apparently used the same spike-in method for both samples, and the exponential relationship between DNA concentration and Cq values, the spiked water and the spiked soil samples showed considerable variation in terms of lipL32 detection, coming mostly from L. interrogans added as control. The spike-in method needs to be standardized and/or the final concentration of bacteria in each spiked sample needs to be better clarified (see comments above).

The reviewer is correct. This type of observations are expected considering the variations in the presence of PCR inhibitors more in soil vs. water samples. We are not looking at the detection limit, which we believe is a false concept when PCR on environmental samples are performed. Adding a spiked sample from the beginning of processing increases our confidence in the potential for our method for detecting *Leptospira.* The concentration mentioned was the final concentration.

- Lines 250-251 “Overall, EMJH cultures with 5-FU or STAFF were favorable for Leptospira growth for the water test group. For the soil test samples, the culture results were more variable”. Yes, but with some differences. EMJH + 5-FU reaches the higher amount of Leptospira-like microorganisms in the second week, while EMJH + STAFF in the third week. After the second week, the bacterial load in EMJH + 5-FU decreases. No bar graphs are shown for the soil samples. Was the bacterial growth evaluated more than once or is it a single tube measurement? From which combination, EMJH + 5-FU or EMJH + STAFF was the DNA extracted and then sequenced?

We performed a single tube experiment for each condition. These cultures were the ones that were then DNA extracted and sequenced. The results are added to the supplementary material.

Microbial composition profiling and Leptospira classification from 16S dataset

-	Lines 270-271 “A very low number of reads were classified under the phylum taxon "Spirochaetota" in the water (1 read) and soil (9 reads) samples when 16S rRNA gene sequence dataset was analyzed”. In Supplementary Figure 3, however, panel A showing the phylogenetic tree of the water samples highlights 9 branches, and panel B (soil samples), a single branch. Contrary to what is indicated in the text.

We addressed this error in the revised version. Thank you for noticing this mistake.

Microbial profiling of the enrichment culture

-	Lines 309-310 “It is interesting to note that 98% and 99% of leptospiral reads in the soil and water samples were classified under either a saprophytic species or an unclassified species”. I do not think this is surprising, as the enrichment was done over a 4-week period, and saprophytic Leptospira has a shorter doubling time than pathogenic Leptospira (approximately 8h versus 20h). It would be interesting to take samples for sequencing at different time points (one per week, for example, or after 2 and 4 weeks) and compare the distribution between pathogenic and saprophytic leptospires over time.

- It would have been interesting to compare to low "activity" sample that could serve as a negative control.

- The sample have been pooled, what would have been the results without pooling them?. Is this representative or is there only one sample that account for all the results?.

We are not sure what the reviewer meant by low “activity” sample. Samples came from a single location, and pooling may not make any difference in the results. If the sites were different, further testing of individual samples would have a more significant impact.
Results
-Does the analysis presented match the analysis plan?
-Are the results clearly and completely presented?
-Are the figures (Tables, Images) of sufficient quality for clarity?

Reviewer #3: In the present study, Gorman and colleagues apply different approaches to identify Leptospira spp. in environmental samples, i.e., soil and water. The methods assessed include PCR, direct metagenomic sequencing, and culture with subsequent PCR or metagenomic sequencing. The results obtained highlight the advantages of using culture enrichment and then PCR/sequencing, as opposed to direct PCR or 16S-based metagenomics. While the authors underline as a novelty the fact that enriched cultures are more suitable for detecting abundance and diversity in environmental samples, this is not surprising. Leptospira is slower growing than other bacteria present in the environment, so an enrichment would certainly be helpful for its detection in a complex sample. However, the impact of the article resides in the comparison of the most commonly used techniques such as direct PCR, or 16S-based metagenomics of samples, versus traditional enrichment methods along with ONT
Nanopore sequencing, in detecting Leptospira spp. They even test several culture media and antimicrobial agents to ensure Leptospira growth. Being aware of the advantages of Nanopore sequencing (short-term, cost-effective sample processing), this sounds promising as an epidemiological surveillance tool. 
Please not that the sequencing data was not availlable.

We thank the reviewer for acknowledging the importance of this work and for positive critiques which have helped us improve our manuscript.

We have uploaded the data.
