# Supplementary material for: Leptospira enrichment culture followed by ONT metagenomic sequencing allows better detection of Leptospira presence and diversity in water and soil samples
Source: PLoS Negl Trop Dis. 2022 Oct 26;16(10):e0010589. doi: 10.1371/journal.pntd.0010589 (PMC9639851; doi:10.1371/journal.pntd.0010589)
Supplement: Attachment — Submitted filename: Reply_to_Reviewer_Comments_R2.docx [file pntd.0010589.s005.docx]

**Reply to reviewers’ comments**

We thank reviewers 1 for the time and effort it took to review our manuscript and providing valuable suggestions. All the comments from reviewer 3 is addressed in the revised version

- The 23S-based PCR is still a bit problematic. Lines 391-392 specifically states that the primers used amplify non-pathogenic Leptospira. However, 23S is detected in the “water spiked” sample inoculated with pathogenic L. interrogans, but not in the “water test” sample. I wondered if “water test” and “soil test” had been interchanged, which is also supported by the fact that 23S in both “soil test” and “water spiked” have comparable Cq (considering the associated error). I understand that the authors had used controls during sample processing and ruled out such an exchange. So, if there was not a DNA swap when doing PCR, the 23S-based PCR is not valid and not worthy to be shown.

Based on reviewer’s criticism, we removed the 23s PCR from the manuscript and revised the text, figures and references.

- Figure 2 and 3 lists L. macculloughii among the detected species. Although L. macculloughii was described in Thibeaux 2018, it was later not considered again (Vincent et al., 2019), since it was determined to be the result of a mixed culture between L. meyeri and L. levetti.

We were infact going to make a comment on this and then missed it while preparing the manuscript. Thank you for pointing this out. In the revised version we removed this species from the figures and added a note to the phylogenetic tree legend.

Minor comments/suggestions:

- Lines 284-285. Differences in font type.
- Lines 313 and 354. Leptospira should be in italics.
- Lines 315-316. It is mentioned that two additional P1 species were found in water samples compared to soil samples. From Figure 3C it is not so evident which were these two species, so it might be worth mentioning them in the text.
- Line 316. “Figure 4C” is cited but does not exist in this version, so it should be changed to “Figure 3C”. Also, the caption in Figure 3 contains cross-references to Figure 4 from the previous version.
- Line 366. Punctuation marks: two consecutive full stops at the end of a sentence.

We went through the manuscript carefully and addressed all these commnets. We apologize for these silly errors which could have been avoided. Thank you for helping us to improve our manuscript.
